# Midlife brain metastases in the United States: Is male at risk?

**DOI:** 10.1002/cam4.4499

**Published:** 2022-01-12

**Authors:** Wenqiang Che, Yujiao Wang, Xiangyu Wang, Jun Lyu

**Affiliations:** ^1^ Department of Neurosurgery The First Affiliated Hospital of Jinan University Guangzhou China; ^2^ Department of Clinical Research The First Affiliated Hospital of Jinan University Guangzhou China; ^3^ Department of Pathology Xuanwu Hospital, Capital Medical University Beijing China

**Keywords:** brain metastases, female, male, midlife, survival

## Abstract

**Background:**

Population‐based estimates of the impact of gender throughout the whole course of brain metastases (BMs) at the time of diagnosis of systemic malignancies are insufficient. We aimed to discover the influence of gender on the presence of BMs in newly diagnosed malignancies and the survival of those patients on a population‐based level.

**Methods:**

Midlife patients (40 years ≤ age ≤60 years) with newly diagnosed malignancies and BMs at the time of diagnosis were abstracted from the Surveillance, Epidemiology, and End Results (SEER) database of the National Cancer Institute. Clinical variables adjusted patient data. The LASSO regression was performed to exclude the possibility of collinearity. Univariable and multivariable logistic regression analyses were applied to find independent predictors for the presence of BMs, while univariable and multivariable Cox proportional hazard regression analyses were used to determine prognosticators of survival. K‐M curves were used to perform the survival analysis.

**Result:**

276,327 population‐based samples met inclusion criteria between 2014 and 2016, and 5747 (2.08%) patients were diagnosed with BMs at the time of diagnosis of systematic malignancies. Among all midlife patients with cancer, 44.02% (121,634) were male, while 51.68% (2970) were male among patients with BMs at the time of diagnosis. The most frequent tumor type was breast cancer (23.11%), and lung cancer had the highest incidence proportion of BMs among the entire cohort (19.34%). The multivariable logistic regression model suggested that female (vs. male, odds ratio [OR] 1.07, 95% CI: 1.01–1.14, *p* < 0.001) was associated with a higher risk of the presence of BMs at the time of diagnosis. Moreover, in the multivariable Cox model for all‐cause mortality in individuals with BMs at diagnosis, female (vs. male, hazard ratio [HR], 0.86, 95% CI, 0.80–0.92, *p* < 0.001) was shown to have a lower risk of decreased all‐cause mortality.

**Conclusion:**

The middle‐aged females were at increased risk of developing BMs, while the middle‐aged males with BMs were at higher risk of having poorer survival.

## Graphical abstract

Our study is based on a large population derived from the Surveillance, Epidemiology, and End Results (SEER) database. The study found that the middle‐aged females were at a higher risk of developing brain metastases, while the middle‐aged males with brain metastases were at a higher risk of having poorer survival.

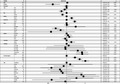

## 1 INTRODUCTION

Statistically, about 20% of cancer patients suffer from brain metastases (BMs).^1^, ^2^, ^3^ This ratio increases with age to around 40% of those aged ≥18.^2^, ^4^, ^5^ However, the actual prevalence can be higher because these estimates are usually limited to those who are being assessed for therapy. Routine brain MRI examinations are not recommended for the majority of cancer patients who do not have neurological symptoms. These tumors are expected to be up to 10 times more prevalent than primary malignant brain tumors.^5^, ^6^, ^7^, ^8^, ^9^, ^10^, ^11^ There has been only one published study^12^ to date highlighting that the incidence of patients with newly diagnosed systemic malignancy with BMs is about 2%. Most BMs metastasize from lung cancer (40%–50%), breast cancer (15%–25%), and melanoma (5%–20%).^3^, ^13^, ^14^, ^15^ Moreover, especially for melanoma, a disposition to metastasize is a very early event.^16^ Headaches, nausea, tiredness, anorexia, affective disturbance, abnormal mental status, cognitive impairment, insomnia, epilepsy, and focal neural function deficits are all common symptoms in individuals with BMs.^17^, ^18^

Multimodal systemic therapies, including a combination of surgical removal, radiotherapy, chemotherapy, immunotherapy, and molecularly targeted therapies, are applied to extend overall survival after being diagnosed with BMs.^1^, ^19^, ^20^, ^21^, ^22^ However, prognosis in cancer patients with BMs remains poor, with a relatively low median survival (2.9 months in newly diagnosed malignancies)^23^ and 2‐year survival rate (8%).^24^ Mounting evidence indicates that gender is associated with the survival of the majority of neoplastic diseases.^25^, ^26^, ^27^, ^28^, ^29^, ^30^, ^31^ Moreover, numerous studies have shown the male gender as an independent risk factor for shorter survival in BMs patients.^4^, ^12^, ^23^ In contrast, the occurrence of BMs has been neglected in long‐term clinical practices, and little research could elaborate on whether the male gender is a risk factor throughout the disease (BMs) course.

The purpose of this work was to apply the Surveillance, Epidemiology, and End Results (SEER) database to discover the influence of gender on the presence of BMs at newly diagnosed malignancies and survival of those patients on a population‐based basis.

## 2 METHODS

Our institutional review board gave their approval to this project, and the committee waived the requirement for informed consent.

### 2.1 Study design and population

**FIGURE 1:**
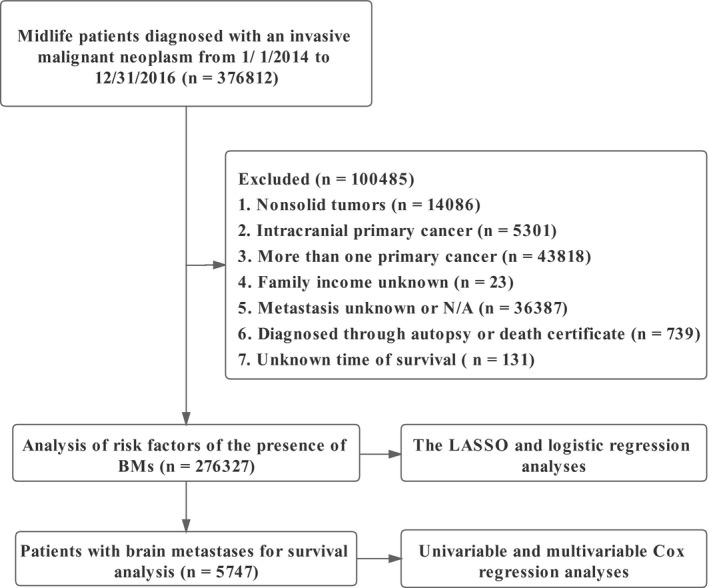
The workflow of the study

The National Cancer Institute (NCI)‐sponsored SEER program collected information from 34.6% of cancer patients in the United States and published information on demographics, disease, clinicopathological features, and therapeutic procedure‐related covariates online.^32^, ^33^ We accessed the Surveillance Research Program, released on April 2019, using SEER*Stat (version 8.3.9) and identified 376,812 midlife patients diagnosed with an invasive malignant neoplasm from January 1, 2014, to December 31, 2016. Midlife was recognized as being between the ages of 40 (≥40) and 60 (≤60).^34^, ^35^ The presence or absence of BMs was verified prior to treatment. We excluded patients: (1) with nonsolid tumors; (2) with intracranial primary cancer; (3) diagnosed with more than one primary cancer; (4) with family income unknown; (5) with extracranial organ metastasis unknown or not applicable; (6) diagnosed through autopsy or death certificate; (7) with survival time unknown, 276,327 (73.32%) patients included in the final cohort for analysis. The workflow is shown in Figure 1. We divided patients into two groups based on their gender. For patients with malignancy who had BMs at the time of diagnosis, absolute numbers and incidence proportions were computed; incidence proportions were determined as well after stratification by years at diagnosis, race, regions, marital status, insurance, lymph nodal positive rate (LNPRate), the number of extracranial organs involved (only bone, liver, and lung were included) by metastases (0, 1, 2, and 3 represent the number of organs affected), and the cancer type. Histology, rather than the primary site, was used to identify patients with sarcoma and melanoma. The location of the tumor and its histology were identified using ICD‐O‐3 codes (International Classification of Diseases for Oncology, Third Edition). The nodal staging was replaced by LNPRate, which was progressed in increments of 20%. According to the SEER database, race/ethnicity was classified as non‐Hispanic white, non‐Hispanic black, non‐Hispanic American Indian/Alaska Native, non‐Hispanic Asian or Pacific Islander, Hispanic, or other. Standard SEER follow‐up methods were used to check on participants’ vital status, death date, and cause of death once a year. The end date for the follow‐up was December 31, 2016. Mortality was defined as death from any cause.

### 2.2 Statistical analysis

The Least absolute shrinkage and selection operator (LASSO), a regularized regression approach performed using the “glmnet” package, was used to exclude multicollinearity and automated variable selection in the study.^36^, ^37^, ^38^ Univariable and multivariable logistic regression models were applied to determine whether the variables selected from the LASSO regression were associated with the occurrence of BMs at diagnosis. The survival hazard was then assessed using univariate and multivariate Cox proportional hazard regression models.^39^ Finally, survival curves for each variable in the multivariate Cox model with a *p*‐value <0.05 were created using the K‐M analysis and compared using the two‐tailed log‐rank test.^40^, ^41^ The Chi‐squared test was used to compare categorical demographic features, while continuous variables were given as means (standard deviation) and compared using the *t*‐test. Statistical analyses were performed using RStudio based on the R programming language^42^ version 4.0.5 that was released on 2021–03–31 (cran.r‐project.org/src/base/R‐4/).

## 3 RESULT

Baseline demographic and clinical characteristics by gender for patients with cancer (any Stage) and BMs at diagnosis were presented in Table 1. In the 2014–2016 period, 276,327 patients were diagnosed with a solid malignancy, and 5747 patients, 2.08% of all patients, had BMs at diagnosis. A total of 121,634 patients, 44.02% of all patients with malignancy and 51.68% of patients with BMs, were male. Breast cancer was the most frequently‐observed cancer, accounting for 23.1% of cases. Lung cancer, renal cancer, and endometrial cancer ranked as the top three in terms of the incidence proportion of BMs among the entire cohort. There were significant differences across gender for patients with BMs for age, region, insurance status, median household income, the percent of high education, and T stage. The remaining variables, race, year of diagnosis, marital status, laterality of primary malignancy, and the presence of other organ metastases, were not significantly different between the male and female groups.

**TABLE 1:** Baseline characteristics of patients with newly diagnosed systematic malignancies and with brain metastases present at the time of diagnosis

Variables	Categories	Patients with cancer (any stage): *N* (%)	Sex		Patients with brain metastases at diagnosis: *N* (%)	Incidence proportion of brain metastases	Sex		*p* value
			Male	Female			Male	Female	
Age	Continuous	52.84 (5.49)	53.82 (5.10)	52.08 (5.67)	54.12 (4.79)	—	54.46 (4.67)	53.94 (4.90)	<0.001
Year	2014	92072 (33.32%)	40509 (44%)	51563 (56%)	1941 (33.77%)	2.11%	1003 (51.67%)	938 (48.33%)	0.997
	2015	92604 (33.51%)	40785 (44.04%)	51819 (55.96%)	1916 (33.34%)	2.07%	989 (51.62%)	927 (48.38%)	
	2016	91651 (33.17%)	40340 (44.01%)	51311 (55.99%)	1890 (32.89%)	2.06%	978 (51.75%)	912 (48.25%)	
Race	NHW	172474 (62.42%)	78103 (45.28%)	94371 (54.72%)	3858 (67.13%)	2.24%	2017 (52.28%)	1841 (47.72%)	0.260
	NHB	36516 (13.21%)	17909 (49.04%)	18607 (50.96%)	867 (15.09%)	2.37%	439 (50.63%)	428 (49.37%)	
	NHAI/AN	2000 (0.72%)	842 (42.1%)	1158 (57.9%)	44 (0.77%)	2.2%	23 (52.27%)	21 (47.73%)	
	NHAPI	22309 (8.07%)	7378 (33.07%)	14931 (66.93%)	457 (7.95%)	2.05%	213 (46.61%)	244 (53.39%)	
	Hispanic	38914 (14.08%)	15081 (38.75%)	23833 (61.25%)	508 (8.84%)	1.31%	272 (53.54%)	236 (46.46%)	
	Others	4114 (1.49%)	2321 (56.42%)	1793 (43.58%)	13 (0.23%)	0.32%	6 (46.15%)	7 (53.85%)	
Region	Northeast	44115 (15.96%)	19471 (44.14%)	24644 (55.86%)	909 (15.82%)	2.06%	433 (47.63%)	476 (52.37%)	0.001
	Midwest	25277 (9.15%)	11607 (45.92%)	13670 (54.08%)	650 (11.31%)	2.57%	328 (50.46%)	322 (49.54%)	
	South	68520 (24.8%)	32435 (47.34%)	36085 (52.66%)	1773 (30.85%)	2.59%	978 (55.16%)	795 (44.84%)	
	West	138415 (50.09%)	58121 (41.99%)	80294 (58.01%)	2415 (42.02%)	1.74%	1231 (50.97%)	1184 (49.03%)	
Marital	Married	154678 (55.98%)	67734 (43.79%)	86944 (56.21%)	2710 (47.16%)	1.75%	1389 (51.25%)	1321 (48.75%)	0.543
	Others	121649 (44.02%)	53900 (44.31%)	67749 (55.69%)	3037 (52.84%)	2.5%	1581 (52.06%)	1456 (47.94%)	
Insurance	Insured	206281 (74.65%)	88950 (43.12%)	117331 (56.88%)	3625 (63.08%)	1.76%	1810 (49.93%)	1815 (50.07%)	<0.001
	Others	70046 (25.35%)	32684 (46.66%)	37362 (53.34%)	2122 (36.92%)	3.03%	1160 (54.67%)	962 (45.33%)	
Primary site	Head & Neck	14508 (5.25%)	10912 (75.21%)	3596 (24.79%)	41 (0.71%)	0.28%	35 (85.37%)	6 (14.63%)	—
	Thyroid	15951 (5.77%)	3829 (24%)	12122 (76%)	14 (0.24%)	0.09%	7 (50%)	7 (50%)	
	Lung	23343 (8.45%)	12201 (52.27%)	11142 (47.73%)	4514 (78.55%)	19.34%	2332 (51.66%)	2182 (48.34%)	
	Breast	63860 (23.11%)	372 (0.58%)	63488 (99.42%)	270 (4.7%)	0.42%	5 (1.85%)	265 (98.15%)	
	Colorectal	29787 (10.78%)	16690 (56.03%)	13097 (43.97%)	95 (1.65%)	0.32%	49 (51.58%)	46 (48.42%)	
	Kidney	13949 (5.05%)	9228 (66.16%)	4721 (33.84%)	222 (3.86%)	1.59%	165 (74.32%)	57 (25.68%)	
	Melanoma	4264 (1.54%)	2635 (61.8%)	1629 (38.2%)	16 (0.28%)	0.38%	8 (50%)	8 (50%)	
	Liver	7940 (2.87%)	5742 (72.32%)	2198 (27.68%)	38 (0.66%)	0.48%	26 (68.42%)	12 (31.58%)	
	Ovarian	13885 (5.02%)	0 (0%)	13885 (100%)	29 (0.5%)	0.21%	0 (0%)	29 (100%)	
	Endometrial	3814 (1.38%)	0 (0%)	3814 (100%)	17 (0.3%)	4.46%	0 (0%)	17 (100%)	
	Prostate	33370 (12.08%)	33370 (100%)	0 (0%)	17 (0.3%)	0.51%	17 (100%)	0 (0%)	
	Others	51656 (18.69%)	26655 (51.6%)	25001 (48.4%)	474 (8.25%)	0.71%	326 (68.78%)	148 (31.22%)	
Laterality	Unilateral	271007 (98.07%)	119814 (44.21%)	151193 (55.79%)	5448 (94.8%)	2.01%	2817 (51.71%)	2631 (48.29%)	0.857
	Others	5320 (1.93%)	1820 (34.21%)	3500 (65.79%)	299 (5.2%)	5.62%	153 (51.17%)	146 (48.83%)	
T‐stage	0	911 (0.33%)	470 (51.59%)	441 (48.41%)	157 (2.73%)	17.23%	99 (63.06%)	58 (36.94%)	0.002
	1	116191 (42.05%)	39247 (33.78%)	76944 (66.22%)	626 (10.89%)	0.54%	311 (49.68%)	315 (50.32%)	
	2	65505 (23.71%)	31204 (47.64%)	34301 (52.36%)	1260 (21.92%)	1.92%	607 (48.17%)	653 (51.83%)	
	3	55530 (20.1%)	29597 (53.3%)	25933 (46.7%)	1496 (26.03%)	2.69%	796 (53.21%)	700 (46.79%)	
	4	22127 (8.01%)	11700 (52.88%)	10427 (47.12%)	1648 (28.68%)	7.45%	851 (51.64%)	797 (48.36%)	
	Others	16063 (5.81%)	9416 (58.62%)	6647 (41.38%)	560 (9.74%)	3.49%	306 (54.64%)	254 (45.36%)	
LNPRate	0%−20%	109594 (39.66%)	33420 (30.49%)	76174 (69.51%)	174 (3.03%)	0.16%	85 (48.85%)	89 (51.15%)	0.241
	21%–40%	9795 (3.54%)	2551 (26.04%)	7244 (73.96%)	26 (0.45%)	0.27%	13 (50%)	13 (50%)	
	41%−60%	5502 (1.99%)	1255 (22.81%)	4247 (77.19%)	26 (0.45%)	0.47%	8 (30.77%)	18 (69.23%)	
	61%−80%	2599 (0.94%)	662 (25.47%)	1937 (74.53%)	18 (0.31%)	0.69%	6 (33.33%)	12 (66.67%)	
	81%−100%	6525 (2.36%)	1628 (24.95%)	4897 (75.05%)	155 (2.7%)	2.38%	82 (52.9%)	73 (47.1%)	
	Unexamined	129727 (46.95%)	76058 (58.63%)	53669 (41.37%)	4495 (78.21%)	3.46%	2339 (52.04%)	2156 (47.96%)	
	Others	12585 (4.55%)	6060 (48.15%)	6525 (51.85%)	853 (14.84%)	6.78%	437 (51.23%)	416 (48.77%)	
Involved organs*	0	246374 (89.16%)	105333 (42.75%)	141041 (57.25%)	2462 (42.84%)	1%	1273 (51.71%)	1189 (48.29%)	0.263
	1	21273 (7.7%)	11710 (55.05%)	9563 (44.95%)	1820 (31.67%)	8.56%	966 (53.08%)	854 (46.92%)	
	2	7161 (2.59%)	3818 (53.32%)	3343 (46.68%)	1066 (18.55%)	14.89%	539 (50.56%)	527 (49.44%)	
	3	1511 (0.55%)	773 (50.89%)	746 (49.11%)	399 (6.94%)	26.27%	192 (48.12%)	207 (51.88%)	
Income*	Continuous	0.57 (0.38)	0.77 (0.20)	0.78 (0.20)	0.75 (0.20)		0.74 (0.20)	0.76 (0.20)	0.001
Education*	Continuous	102.29 (79.01)	138.41 (58.10)	138.95 (58.34)	140.45 (58.53)		142.13 (59.08)	138.65 (57.89)	0.024
Total		276327 (100%)	121634 (100%)	154693 (100%)	5747 (100%)	2.08%	2970 (100%)	2777 (100%)	—

Note:

Income*, median household income, increased by per $10 000 annual; Education*, high school education percent, increased by per 10%; Involved organs*, number of involved organs excluding brain metastases.

Abbreviations: NHAI/AN, Non‐Hispanic American Indian/Alaska Native; NHAPI, Non‐Hispanic Asian or Pacific Islander; NHB, Non‐Hispanic Black; NHW, Non‐Hispanic White.

**FIGURE 2:**
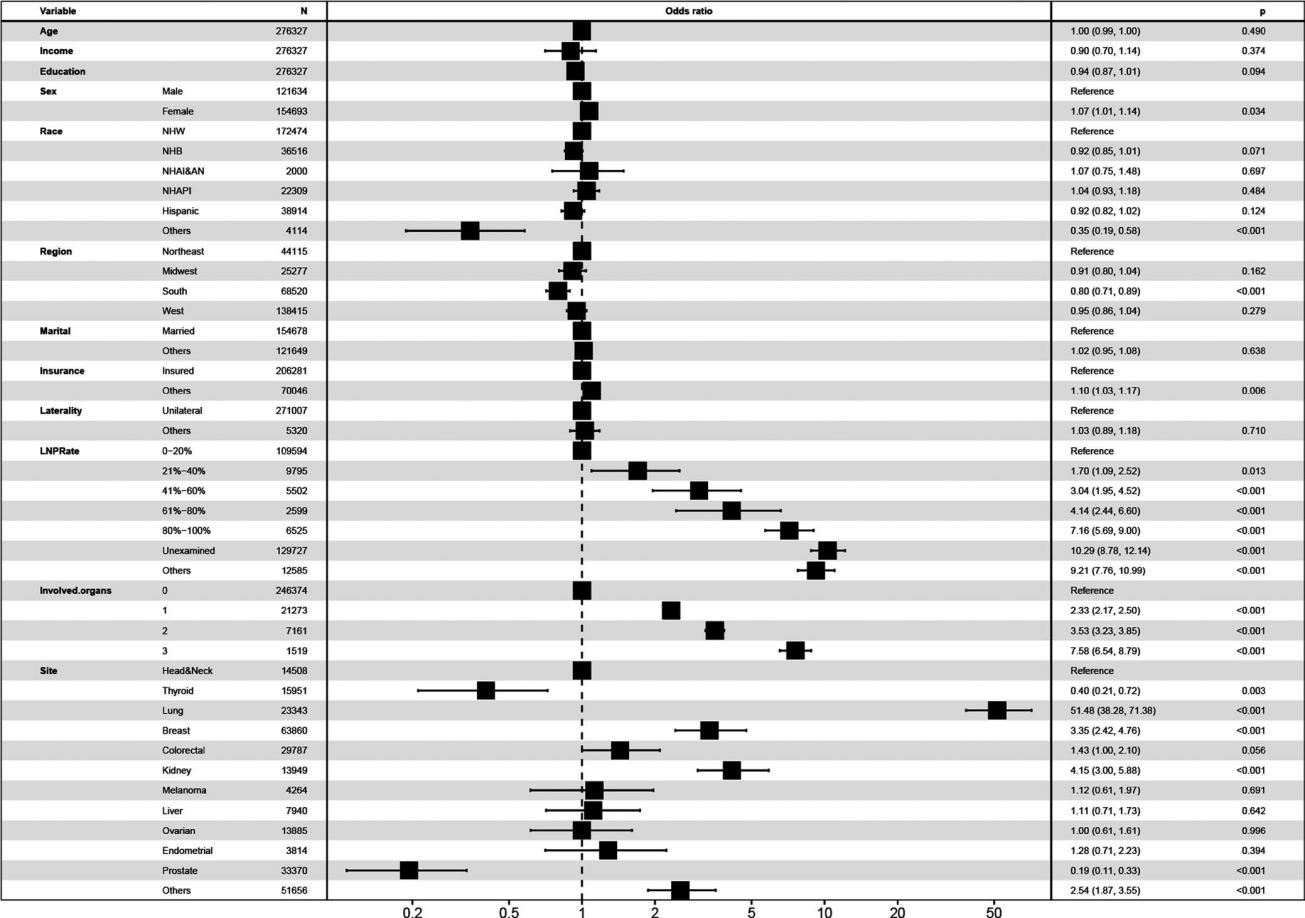
According to multivariate logistic regression analysis, female gender, other insurance status, lymph node‐positive rate (LNPRate) between 21% and 40%, LNPRate between 41% and 60%, LNPRate between 61% and 80%, LNPRate between 81% and 100%, more than one (≥1) extracranial involved organ, and tumor located in the lung, breast, or kidney were all significantly associated with a higher risk of developing brain metastases at the time of diagnosis

In the progression of fitting logistic regression models to data, there is always a concern about over‐fitting. We used LASSO to increase model accuracy and decrease model overfitting. The results of LASSO are summarized in Table 2 and Figure S1. All variables were included in the univariable logistic regression model. According to the multivariable logistic regression model (Figure 2 and Table 3), in the whole cohort, variables that were associated with a substantially increased risk of the presence of BMs at the time of diagnosis were female (vs. male, odds ratio [OR] 1.1, 95% CI: 1.01–1.14, *p *= 0.034), other insurance status (vs. insured, OR 1.10, 95% CI: 1.03–1.17, *p *= 0.006), LNPRate between 21% and 40% (vs. 0%−20%, OR 1.70, 95% CI: 1.12–2.57, *p* < 0.001), LNPRate between 41% and 60% (vs. 0%−20%, OR 3.04, 95% CI: 2.00–4.61, *p* < 0.001), LNPRate between 61% and 80% (vs. 0%−20%, OR 4.14, 95% CI: 2.52–6.79, *p* < 0.001), LNPRate between 81% and 100% (vs. 0%−20%, OR 7.16, 95% CI: 5.69–9, *p* < 0.001), unexamined LNPRate (vs. 0%−20%, OR 10.29, 95% CI: 8.76–12.1, *p* < 0.001), 1 extracranial affected organ (vs. 0, OR 2.33, 95% CI: 2.17–2.5, *p* < 0.001), 2 extracranial affected organs (vs. 0, OR 3.53, 95% CI: 3.23–3.85, *p* < 0.001), 3 extracranial affected organs (vs. 0, OR 7.58, 95% CI: 6.54–8.79, *p* < 0.001), primary tumor located in lung (vs. head & neck, OR 51.48, 95% CI: 37.73–70.24, *p* < 0.001), primary tumor located in breast (vs. head & neck, OR 3.35, 95% CI: 2.39–4.69, *p* < 0.001), and primary tumor located in kidney (vs. head & neck, OR 4.15, 95% CI: 2.97–5.8, *p* < 0.001). High school education percentage and median household income were not related to a risk of the presence of BMs at diagnosis in the multivariable model. South region (vs. Northeast, OR 0.80, 95% CI: 0.71–0.89, *p* < 0.001), primary tumor located in thyroid (vs. head & neck, OR 0.40, 95% CI: 0.22–0.74, *p *= 0.003), and primary tumor located in prostate (vs. head & neck, OR 0.19, 95% CI: 0.11–0.34, *p* < 0.001) were related to marginally lower odds of BMs at diagnosis.

**TABLE 2:** The absolute value of the coefficients in LASSO regression

Variables	Coefficients
Age	0.00073
Income*	0.00414
Education*	0.00093
Sex	0.00214
Year	0.00018
Race	0.00185
Region	0.00097
Marital	0.00123
Insurance	0.00416
Laterality	0.03176
Primary site	0.00471
T‐stage	0.00328
LNPRate	0.00531
Involved organs	0.06312
Intercept	0.11331

Note:

Income*, median household income, increased by per $10 000 annual; Education*, high school education percent, increased by per 10%.

**TABLE 3:** Univariable and multivariable logistic regression models for the presence of brain metastases at diagnosis of systematic malignancies

Variables	Categories	Univariable		Multivariable	
		OR (95%CI)	*p*‐value	OR (95%CI)	*p*‐value
Age		1.05 (1.05,1.06)	<0.001	1.00 (0.99,1.00)	0.491
Sex	Male	Ref.		Ref.	
	Female	0.73 (0.7,0.77)	<0.001	1.07 (1.01,1.14)	0.034
Year	2014	Ref.		—	—
	2015	0.98 (0.92,1.04)	0.505	—	—
	2016	0.98 (0.92,1.04)	0.485	—	—
Race	NHW	Ref.		Ref.	
	NHB	1.06 (0.98,1.14)	0.158	0.92 (0.85,1.01)	0.071
	NHAI/AN	0.98 (0.73,1.33)	0.908	1.07 (0.76,1.5)	0.697
	NHAPI	0.91 (0.83,1.01)	0.070	1.04 (0.93,1.18)	0.484
	Hispanic	0.58 (0.53,0.64)	<0.001	0.92 (0.82,1.02)	0.124
	Others	0.14 (0.08,0.24)	<0.001	0.35 (0.2,0.6)	<0.001
Region	Northeast	Ref.		Ref.	
	Midwest	1.26 (1.13,1.39)	<0.001	0.91 (0.8,1.04)	0.162
	South	1.27 (1.17,1.38)	<0.001	0.8 (0.71,0.89)	<0.001
	West	0.85 (0.79,0.92)	<0.001	0.95 (0.86,1.04)	0.279
Marital	Married	Ref.		Ref.	
	Others	1.44 (1.36,1.51)	<0.001	1.02 (0.95,1.08)	0.638
Insurance	Insured	Ref.		Ref.	
	Others	1.74 (1.65,1.84)	<0.001	1.1 (1.03,1.17)	0.006
Primary Site	Head & Neck	Ref.		Ref.	
	Thyroid	0.31 (0.17,0.57)	<0.001	0.4 (0.22,0.74)	0.003
	Lung	84.59 (62.15,115.13)	<0.001	51.48 (37.73,70.24)	<0.001
	Breast	1.5 (1.08,2.08)	0.016	3.35 (2.39,4.69)	<0.001
	Colorectal	1.13 (0.78,1.63)	0.517	1.43 (0.99,2.07)	0.056
	Kidney	5.71 (4.09,7.97)	<0.001	4.15 (2.97,5.8)	<0.001
	Melanoma	1.33 (0.75,2.37)	0.335	1.12 (0.63,2.01)	0.691
	Liver	1.7 (1.09,2.64)	0.019	1.11 (0.71,1.73)	0.642
	Ovarian	0.74 (0.46,1.19)	0.212	0.9987 (0.6184,1.6127)	0.996
	Endometrial	1.58 (0.9,2.78)	0.114	1.28 (0.72,2.27)	0.394
	Prostate	0.18 (0.1,0.32)	<0.001	0.19 (0.11,0.34)	<0.001
	Others	3.27 (2.37,4.5)	<0.001	2.54 (1.84,3.5)	<0.001
Laterality	Unilateral	Ref.		Ref.	
	Others	2.92 (2.59,3.29)	<0.001	1.03 (0.89,1.18)	0.71
T‐stage	0	Ref.		—	—
	1	0.93 (0.72,1.2)	0.572	—	—
	2	0.94 (0.72,1.22)	0.638	—	—
	3	0.94 (0.72,1.22)	0.655	—	—
	4	1.01 (0.77,1.32)	0.961	—	—
	Others	0.90 (0.69,1.17)	0.418	—	—
LNPRate	0%−20%	Ref.		Ref.	
	21%–40%	1.68 (1.11,2.54)	0.014	1.7 (1.12,2.57)	<0.001
	41%−60%	2.88 (1.89,4.38)	<0.001	3.04 (2,4.61)	<0.001
	61%−80%	4.4 (2.71,7.16)	<0.001	4.14 (2.52,6.79)	<0.001
	81%−100%	15.36 (12.35,19.1)	<0.001	7.16 (5.69,9)	<0.001
	Unexamined	22.54 (19.37,26.23)	<0.001	10.29 (8.76,12.1)	<0.001
	Others	45.45 (38.57,53.56)	<0.001	9.21 (7.74,10.96)	<0.001
Involved organs	0	Ref.		Ref.	
	1	9.32 (8.76,9.92)	<0.001	2.33 (2.17,2.5)	<0.001
	2	17.39 (16.11,18.77)	<0.001	3.53 (3.23,3.85)	<0.001
	3	35.44 (31.4,40.01)	<0.001	7.58 (6.54,8.79)	<0.001
Income*		0.47 (0.41,0.54)	<0.001	0.9 (0.7,1.14)	0.373
Education*		1.05 (1,1.09)	0.042	0.94 (0.87,1.01)	0.094

Note:

Income*, median household income, increased by per $10 000 annual; Education*, high school education percent, increased by per 10%.

Abbreviations: NHAI/AN, Non‐Hispanic American Indian/Alaska Native; NHAPI, Non‐Hispanic Asian or Pacific Islander; NHB, Non‐Hispanic Black; NHW, Non‐Hispanic White.

**FIGURE 3:**
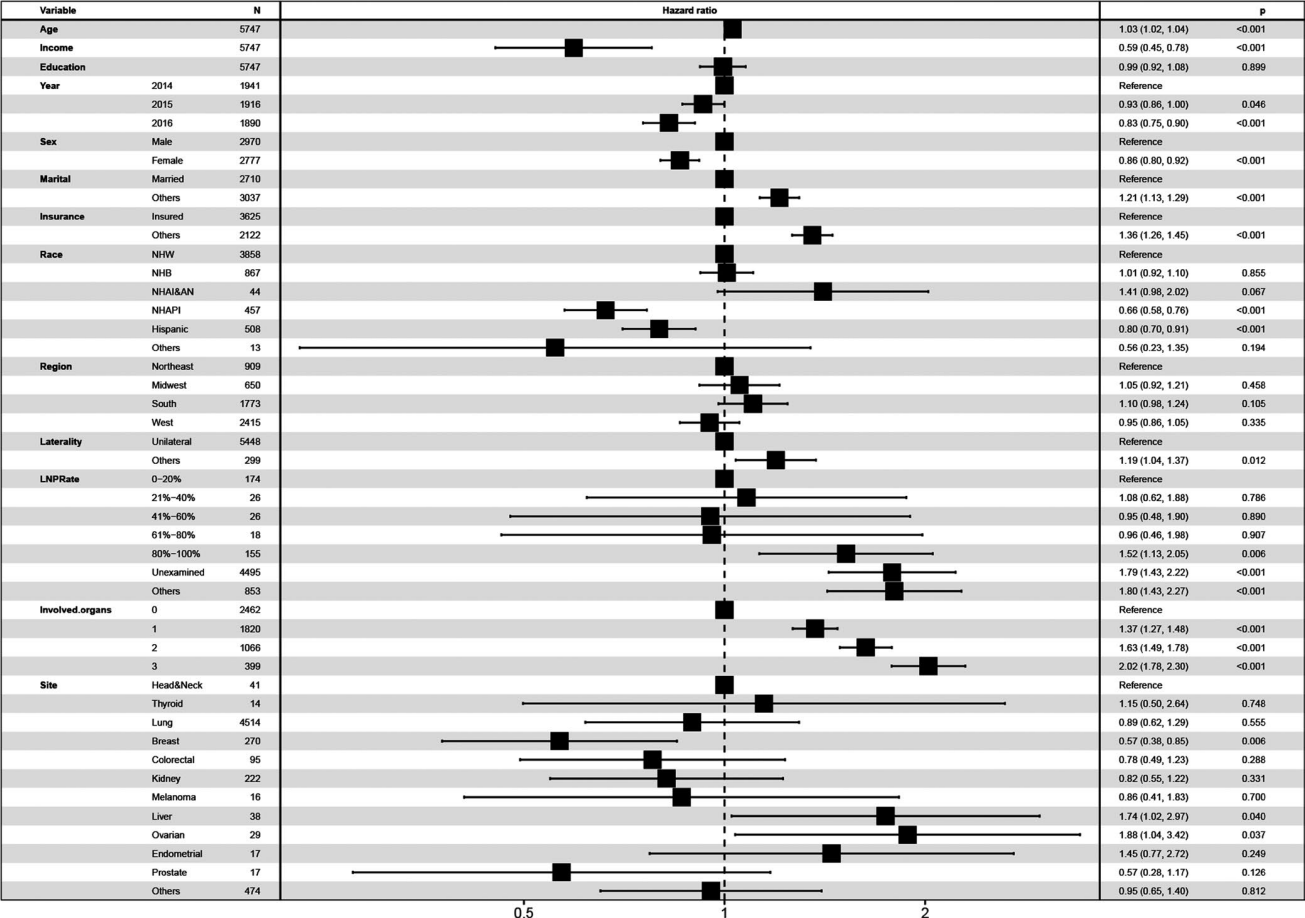
The Cox proportional hazards model, including all possible risk factors, indicated that year of diagnosis at 2015 and 2016, the increased median household income, female gender, NHAPI (Race), Hispanic (Race), and tumor located in the breast were significantly associated with improved survival

The Cox proportional hazard models are shown in Figure 3 and Table 4. In the multivariable Cox model, female (vs. male, hazard ratio [HR] 0.86, 95% CI, 0.80–0.92, *p* < 0.001), median household income (increased by per $10,000 annual, HR 0.59, 95% CI, 0.45–0.78, *p* < 0.001), diagnosis at 2015 (vs. 2014, HR 0.93, 95% CI, 0.86–1.00, *p *= 0.046), diagnosis at 2016 (vs. 2014, HR, 0.83, 95% CI, 0.75–0.90, *p* < 0.001), Non‐Hispanic Asian or Pacific Islander (NHAPI) (vs. NHW, HR, 0.66, 95% CI, 0.58–0.76, *p* < 0.001), Hispanic (vs. NHW, HR, 0.80, 95% CI, 0.70–0.91, *p* < 0.001), and primary tumor located in the breast (vs. head & neck, HR 0.57, 95% CI: 0.38–0.85, *p *= 0.006) were significantly associated with a better clinical outcome. Age (increased by per 1, HR, 1.03, 95% CI, 1.02–1.04, *p* < 0.001), other marital status (vs. married, HR 1.21, 95% CI: 1.13–1.29, *p* < 0.001), other insurance status (vs. insured, HR 1.36, 95% CI: 1.26–1.45, *p* < 0.001), other laterality of primary tumor (vs. unilaterality, OR 1.19, 95% CI: 1.04–1.37, *p *= 0.012), LNPRate between 81% and 100% (vs. 0%−20%, HR 1.52, 95% CI: 1.13–2.05, *p *= 0.006), unexamined LNPRate (vs. 0%−20%, HR 1.79, 95% CI: 1.43–2.22, *p* < 0.001), 1 extracranial affected organ (vs. 0, HR 1.37, 95% CI: 1.27–1.48, *p* < 0.001), 2 extracranial affected organs (vs. 0, HR 1.63, 95% CI: 1.49–1.78, *p* < 0.001), 3 extracranial affected organs (vs. 0, HR 2.02, 95% CI: 1.78–2.30, *p* < 0.001), primary tumor located in the liver (vs. head & neck, HR 1.74, 95% CI: 1.02–2.97, *p *= 0.040), and primary tumor located in the ovarian (vs. head & neck, HR 1.88, 95% CI: 1.04–3.42, *p *= 0.037) were significantly related to a shorter survival time.

**TABLE 4:** Univariable and multivariable Cox regression models for all‐cause mortality among patients with brain metastases

Variables	Categories	Univariable		Multivariable	
		HR (95%CI)	*p*‐value	HR (95%CI)	*p*‐value
Age		1.03 (1.02,1.03)	<0.001	1.03 (1.02,1.04)	<0.001
Sex	Male	Ref.		Ref.	
	Female	0.80 (0.75,0.86)	<0.001	0.86 (0.80,0.92)	<0.001
Year	2014	Ref.		Ref.	
	2015	0.95 (0.89,1.02)	0.174	0.93 (0.86,1.00)	0.046
	2016	0.84 (0.76,0.92)	<0.001	0.83 (0.75,0.90)	<0.001
Race	NHW	Ref.		Ref.	
	NHB	1.16 (1.06,1.26)	0.001	1.01 (0.92,1.10)	0.855
	NHAI/AN	1.16 (0.81,1.66)	0.431	1.41 (0.98,2.02)	0.067
	NHAPI	0.59 (0.52,0.68)	<0.001	0.66 (0.58,0.76)	<0.001
	Hispanic	0.82 (0.73,0.93)	0.001	0.80 (0.70,0.91)	<0.001
	Others	0.54 (0.23,1.31)	0.172	0.56 (0.23,1.35)	0.194
Region	Northeast	Ref.		Ref.	
	Midwest	1.21 (1.07,1.37)	0.002	1.05 (0.92,1.21)	0.458
	South	1.35 (1.22,1.48)	<0.001	1.10 (0.98,1.24)	0.105
	West	0.98 (0.89,1.08)	0.724	0.95 (0.86,1.05)	0.335
Marital	Married	Ref.		Ref.	
	Others	1.34 (1.25,1.42)	<0.001	1.21 (1.13,1.29)	<0.001
Insurance	Insured	Ref.		Ref.	
	Others	1.41 (1.32,1.51)	<0.001	1.36 (1.26,1.45)	<0.001
Primary site	Head & Neck	Ref.		Ref.	
	Thyroid	0.76 (0.33,1.73)	0.514	1.15 (0.50,2.64)	0.748
	Lung	0.77 (0.53,1.11)	0.160	0.89 (0.62,1.29)	0.555
	Breast	0.52 (0.35,0.77)	0.001	0.57 (0.38,0.85)	0.006
	Colorectal	0.57 (0.36,0.90)	0.016	0.78 (0.49,1.23)	0.288
	Kidney	0.73 (0.49,1.09)	0.127	0.82 (0.55,1.22)	0.331
	Melanoma	0.53 (0.25,1.12)	0.097	0.86 (0.41,1.83)	0.700
	Liver	1.49 (0.88,2.54)	0.138	1.74 (1.02,2.97)	0.040
	Ovarian	1.33 (0.74,2.40)	0.338	1.88 (1.04,3.42)	0.037
	Endometrial	1.25 (0.67,2.32)	0.489	1.45 (0.77,2.72)	0.249
	Prostate	0.61 (0.30,1.25)	0.178	0.57 (0.28,1.17)	0.126
	Others	0.87 (0.59,1.27)	0.463	0.95 (0.65,1.40)	0.812
Laterality	Unilateral	Ref.		Ref.	
	Others	1.19 (1.03,1.36)	0.015	1.19 (1.04,1.37)	0.012
T‐stage	0	Ref.		—	—
	1	0.99 (0.71,1.38)	0.934	—	—
	2	1.00 (0.71,1.40)	0.991	—	—
	3	0.99 (0.70,1.39)	0.938	—	—
	4	1.01 (0.71,1.43)	0.962	—	—
	Others	0.95 (0.68,1.34)	0.776	—	—
LNPRate	0%−20%	Ref.		Ref.	
	21%–40%	1.00 (0.58,1.73)	0.998	1.08 (0.62,1.88)	0.786
	41%−60%	0.83 (0.42,1.65)	0.591	0.95 (0.48,1.90)	0.890
	61%−80%	0.93 (0.45,1.92)	0.841	0.96 (0.46,1.98)	0.907
	81%−100%	1.50 (1.11,2.02)	0.008	1.52 (1.13,2.05)	0.006
	Unexamined	2.03 (1.64,2.52)	<0.001	1.79 (1.43,2.22)	<0.001
	Others	2.02 (1.60,2.54)	<0.001	1.80 (1.43,2.27)	<0.001
Involved organs	0	Ref.		Ref.	
	1	1.26 (1.16,1.35)	<0.001	1.37 (1.27,1.48)	<0.001
	2	1.41 (1.30,1.54)	<0.001	1.63 (1.49,1.78)	<0.001
	3	1.70 (1.50,1.92)	<0.001	2.02 (1.78,2.30)	<0.001
Income*		0.44 (0.38,0.52)	<0.001	0.59 (0.45,0.78)	<0.001
Education*		1.00 (1.00,1.00)	<0.001	0.99 (0.92,1.08)	0.9**00**

Note:

Income*, median household income, increased by per $10 000 annual; Education*, high school education percent, increased by per 10%.

Abbreviations: NHAI/AN, Non‐Hispanic American Indian/Alaska Native; NHAPI, Non‐Hispanic Asian or Pacific Islander; NHB, Non‐Hispanic Black; NHW, Non‐Hispanic White.

**FIGURE 4:**
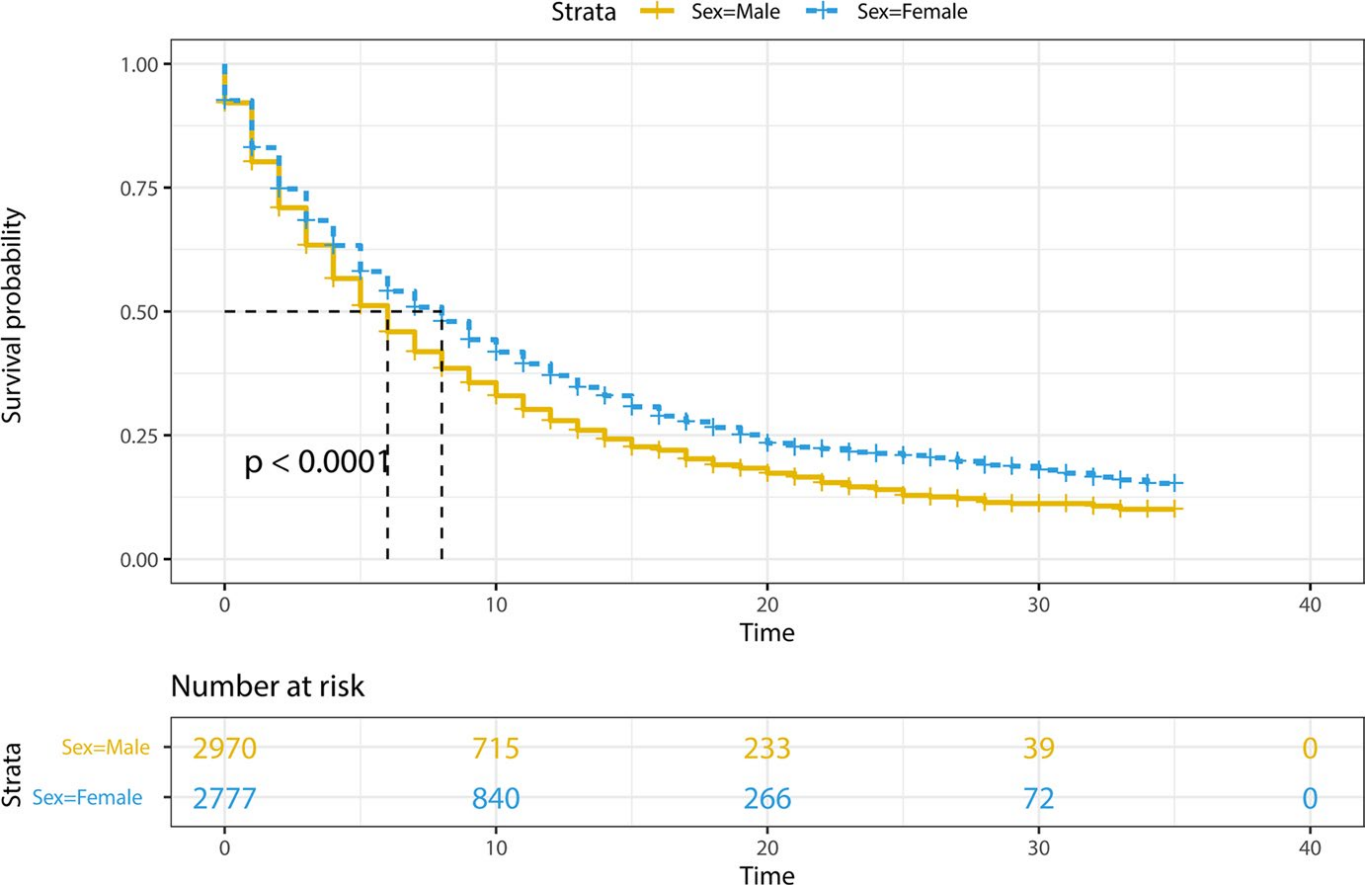
Kaplan–Meier survival curve analysis illustrated that the female gender was associated with increased overall survival among the patients with brain metastases at the time of diagnosis. The median time of survival for the males and females was 6 months and 8 months, respectively

The median survival time in the cohort with BMs at diagnosis, stratified by gender, and the *p*‐values of the log‐rank test in K‐M analysis were present in Table 5. The median survival among the entire male cohort was 6 months, with female patients experiencing the more prolonged median survival (8 months). The female group had a considerably better prognosis, according to the K‐M analysis (*p* < 0.001, Figure 4).

**TABLE 5:** Survival outcomes of patients after diagnosed with brain metastases

Variables	Categories	Median survival time (months, 95% CI)		*p*‐value*
		Male	Female	
Year	2014	5 (4, 6)	7 (6, 8)	<0.001
	2015	6 (5, 7)	8 (7, 9)	<0.001
	2016	7 (6, 8)	9 (8, 10)	0.005
Race	NHW	6 (6, 6)	7 (6, 8)	<0.001
	NHB	5 (4, 6)	6 (5, 7)	0.084
	NHAI/AN	3 (2, 4)	15 (6, 24)	0.018
	NHAPI	11 (8, 14)	15 (11, 19)	0.004
	Hispanic	8 (6, 10)	9 (7, 11)	0.085
	Others	1 (NA, NA)	0 (NA, NA)	0.263
Region	Northeast	7 (6, 8)	9 (7, 11)	0.193
	Midwest	5 (4, 6)	6 (5, 7)	0.013
	South	5 (4, 6)	6 (5, 7)	0.007
	West	6 (5, 7)	10 (9, 11)	<0.001
Marital	Married	7 (6, 8)	9 (8, 10)	<0.001
	Others	5 (5, 5)	6 (5, 7)	<0.001
Insurance	Insured	7 (6, 8)	9 (8, 10)	<0.001
	Others	4 (3, 5)	6 (5, 7)	<0.001
Primary site	Head & Neck	5 (3, 11)	3 (1, 6)	0.412
	Thyroid	4 (2, NA)	—	0.5
	Lung	6 (5, 6)	8 (7, 9)	<0.001
	Breast	12 (8, 21)	12 (9, 15)	0.743
	Colorectal	11 (5, 15)	16 (5, 27)	0.470
	Kidney	6 (4, 10)	5 (3, 11)	0.437
	Melanoma	5 (3, 10)	15 (4, 26)	0.318
	Liver	3 (1, 6)	1.5 (0, NA)	0.380
	Ovarian	—	2 (2, 3)	N/A
	Endometrial	—	4 (3,11)	NA
	Prostate	10 (3, NA)	—	NA
	Others	6 (5, 7)	4 (3, 6)	0.1
Laterality	Unilateral	6 (5, 7)	8 (7, 9)	<0.001
	Others	4 (3, 5)	4 (2, 6)	0.757
T‐stage	0	10 (6, 14)	13 (6, 20)	0.054
	1	9 (7, 11)	11 (9, 13)	<0.001
	2	6 (5, 7)	9 (7, 11)	0.004
	3	6 (5, 7)	7 (6, 8)	0.019
	4	5 (4, 6)	7 (6, 8)	0.002
	Others	4 (3, 5)	5 (3, 7)	0.018
LNPRate	0%−20%	12 (7, 20)	25 (13, 36)	0.093
	21%–40%	18 (15, 20)	13 (9, 19)	0.552
	41%−60%	7 (5, 9)	NA	0.232
	61%−80%	4 (3, NA)	16 (14, NA)	0.020
	81%−100%	6 (3, 9)	14 (10, 18)	0.006
	Unexamined	6 (5, 7)	7 (6, 8)	<0.001
	Others	6 (5, 7)	8 (7, 9)	0.002
Involved organs	0	7 (6, 8)	10 (9, 11)	<0.001
	1	5 (4, 6)	7 (6, 8)	0.001
	2	5 (4, 6)	6 (5, 7)	<0.001
	3	3 (2, 4)	4 (3, 5)	0.010
Total		6 (5. 7)	8 (7, 9)	<0.001

Note:

*p*–value* refers to the log‐rank test.

Abbreviations: CI, confidence interval; NA, not applicable; NHAI/AN, Non‐Hispanic American Indian/Alaska Native; NHAPI, Non‐Hispanic Asian or Pacific Islander; NHB, Non‐Hispanic Black; NHW, Non‐Hispanic White.

## 4 DISCUSSION

In this large population‐based study, we analyzed the relationship between gender and the occurrence of midlife BMs, as well as the prognosis of patients with newly diagnosed extracranial solid malignancy. To the best of our knowledge, this is an advanced epidemiologic study in the United States exploring the impact of gender on BMs utilizing the SEER database. We discovered seven indicators associated with the incidence proportion of BMs: insurance status, race, geographical region, laterality, LNPRate, the number of extracranial involved organs, primary tumor location. Further survival investigation identified eleven prognosticators related to the prognosis of BMs patients. Given that consensus guidelines for patients with systematic malignancy do not recommend screening imaging of the brain, the actual number of BMs patients is likely more than initially thought. Moreover, the poor survival of BMs highlights the importance of very early detection. Our findings are highly geographically generalizable in the United States because the NCI‐SEER program covers over 28% of the U.S. population, underlining the considerable potential value for providers, health care systems, and policy efforts in handling BMs.

Previous studies concerning the molecular subtype of the primary malignant neoplasm could infer potential explanations for some of the presence of BMs. For example, breast cancer in females with epidermal growth factor receptor 2 (EGFR‐2, or HER‐2), positive with or without HR‐positive or triple‐negative subtypes (estrogen receptor (ER) negative, progesterone receptor (P.R.) negative, and normal HER2 levels) is associated with an increased odds ratio for developing BMs than those with ERpositive and/or PRpositive breast cancer.^43^ The increased proclivity of ALK altered nonsmallcell lung cancer to particularly metastasis to the brain is another example of molecular subtype affecting metastatic patterns.^44^ Furthermore, research in the context of epidemiology showed that other extracranial diseases, including metastatic liver or lung involvement, are likely to relate to the detection of BMs at an initial cancer diagnosis.^45^ These findings are in excellent agreement with our results. The greater the involvement of metastases in organ sites (including the liver, lungs, and bone), the greater the risk of developing BMs. Furthermore, we investigated the LNPRate and discovered that when LNPRates rose, the probability of BM presence rose as well. These findings have major clinical implications for detecting BMs early in the course of neurological symptoms utilizing MR scanning.

In 2016, the SEER program first released the data from 2010 to 2013 on the presence or absence of BMs. The following year, Cagney et al.^12^ reported the first and largest epidemiologic study on the incidence proportion of adult patients with BMs based on the SEER database. Their analysis identified the highest rates of BMs in lung cancer patients, and another type of malignancy with an incidence proportion of identified BMs of more than 10% was melanoma and renal cancer. These findings have substantial implications for clinical practice. Furthermore, the work made an effort to identify eight clinicopathological variables related to BMs at diagnosis as well, and gender was not a significant indicator (*p* = 0.49) in multivariable logistic regression analysis.

In contrast, a review^1^ published by *NATURE Review* in 2019 actively demonstrated that in addition to tumor source, molecular subtype, race, age, and geographic location, gender was associated with the development of BMs as well. Previous research found that men had higher BM rates than women (9.7 vs. 7.1 per 100,000 population), which was attributed to a higher incidence of lung primary in men.^46^ The article has been cited more than 700 times, and could it prove that male was at risk of the presence of BMs? Indeed, univariate logistic regression analysis in the study revealed that female gender (vs. male; OR, 0.73; 95% CI, 0.70–0.77; *p* < 0.001) was a significant protective factor for developing midlife BMs. Conversely, when adjusted by other ten significant variables, the multivariate logistic regression analysis illustrated that the female gender (vs. male; OR, 1.07; 95% CI, 1.01–1.14, *p* = 0.034) was at risk of occurrence of BMs. In particular, we recognized the results were the outcome of all variable interactions. The choice of a specific age subgroup, full consideration of social status and clinicopathological data, and a plausible model are crucial for uncovering the disruptive finding. In our view, the results of previous studies were possibly not applicable for midlife patients because features could be overshadowed by the overall characteristics to degrees. When considering breast cancer is one of the more common diseases particularly among patients in midlife, primary tumor site was taken into logistic regression analysis as well, and based on our results, we propose that the female gender is more susceptible to developing BMs in midlife.

During the construction of the outcome predictor, serval prognosticators were used, such as age, the extent of primary disease control, Karnofsky Performance Status, treatment status, and Graded Prognostic Assessment.^1^ Furthermore, an additional study in 2020 raised five prognostic factors for elderly patients with BMs at diagnosis associated with a more favorable outcome.^23^ Those indicators are female gender (vs. male; HR, 0.89; 95% CI, 0.82–0.97; *p* = 0.005), higher median household income, CNS‐directed stereotactic radiation, resection of metastatic tumor, and systemic therapy.^23^ A potential strength is the available information of SEER‐Medicare data and confirmation of the protective effects of female gender (vs. male; HR, 0.90; 95% CI, 0.84–0.95; *p* < 0.001) in metachronous BMs. Our findings are similar to previous research and can be used to supplement previous work. Unfortunately, Cagney et al.^13^ did not analyze the differences in survival stratified by gender among the entire cohort with BMs at diagnosis. Nevertheless, recognizing that breast cancer was the most common cancer among patients in the present study population, we argue that the female gender is associated with a better outcome in patients older than 40 years with BMs at diagnosis.

While the mechanisms by which socioeconomic status influences cancer mortality are likely complicated and indirect,^47^, ^48^, ^49^ research suggests that lower socioeconomic levels (e.g., housing insecurity, financial pressure, and restricted mobility) may affect health, healthcare delivery, and subsequent survival.^50^, ^51^ It is conceivable that a lack of financial support plays a significant role in this population's underutilization of healthcare and specialist cancer services. Another reason may be that patients with low socioeconomic levels have an unbalanced diet, uncontrolled diabetes, and a higher risk of alcoholism and cigarette addiction, all of which are linked to inflammation and could be a trigger for an aberrant immunological response. According to Lamba et al,^23^ having a higher median household income was associated with a longer survival time in senior patients with BMs present at the time of primary cancer diagnosis and in elderly patients with BMs discovered after a primary cancer diagnosis. Our study permitted a similar finding. Further, we found that male patients’ county‐level median household income was significantly lower than female patients, which may provide further explanation for why male patients were related to poorer clinical outcomes.

### 4.1 Limitations

It is crucial to consider the context of the limitations. First, the study was constructed based on the SEER program with regional limitations because the SEER collected patients’ data from 18 cancer registries across the United States.^52^ As a result, extrapolating the findings of our study to the worldwide population of patients with BMs should be done with caution. Second, we were unable to identify patients who develop brain metastases after an initial diagnosis because SEER does not provide information regarding disease recurrence. We considered only synchronous BMs. Third, the number, location, and size of the BMs were not recorded by the SEER program. Third, not all cancers are subjected to screening. As a result, the proportion of unscreened populations with brain metastases is likely to be underestimated. Finally, education level and median household income were specified at the county level rather than the patient level, influencing the logistic and Cox regression results.

## 5 CONCLUSIONS

Despite these limitations, the research provided insight into the risk of developing brain metastases and its prognosis in patients with newly diagnosed malignancies in the United States. The results of our study illustrated that middle‐aged females were at higher risk of developing brain metastases, while middle‐aged males with brain metastases were at risk of having poorer survival. Taken together, our study has important implications for the early identification of high‐risk individuals, individualized therapies, and future trial designs for brain metastases in newly diagnosed malignancies.

## CONFLICT OF INTEREST

There are no conflicts of interest reported by the authors in relation to this paper.

## AUTHOR CONTRIBUTIONS

Dr. Wenqiang Che and Prof. Xiangyu Wang had full access to all the current data in the study and were responsible for the completeness of the data and the accuracy of the result. Wenqiang Che involved in study concept and design, and drafting of the manuscript. Dr. Yujiao Wang, Prof. Jun Lyu and Xiangyu Wang carried out critical revision of the manuscript for necessary. Wenqiang Che and Xiangyu Wang carried out statistical analysis. Jun Lyu and Xiangyu Wang involved in supervision. All the authors carried out acquisition, analysis, or interpretation of data.

## Supporting information

Fig S1

## Data Availability

The raw datasets for this study can be found in the Surveillance, Epidemiology, and End Results Program [https://seer.cancer.gov].
