# Supplementary material for: Midlife brain metastases in the United States: Is male at risk?
Source: Cancer Med. 2022 Jan 12;11(4):1202–16. doi: 10.1002/cam4.4499 (PMC8855893; doi:10.1002/cam4.4499)
Supplement: Supplementary file 1 — Fig S1 [file CAM4-11-1202-s001.docx]

Supplementary Material

## Supplementary Figures


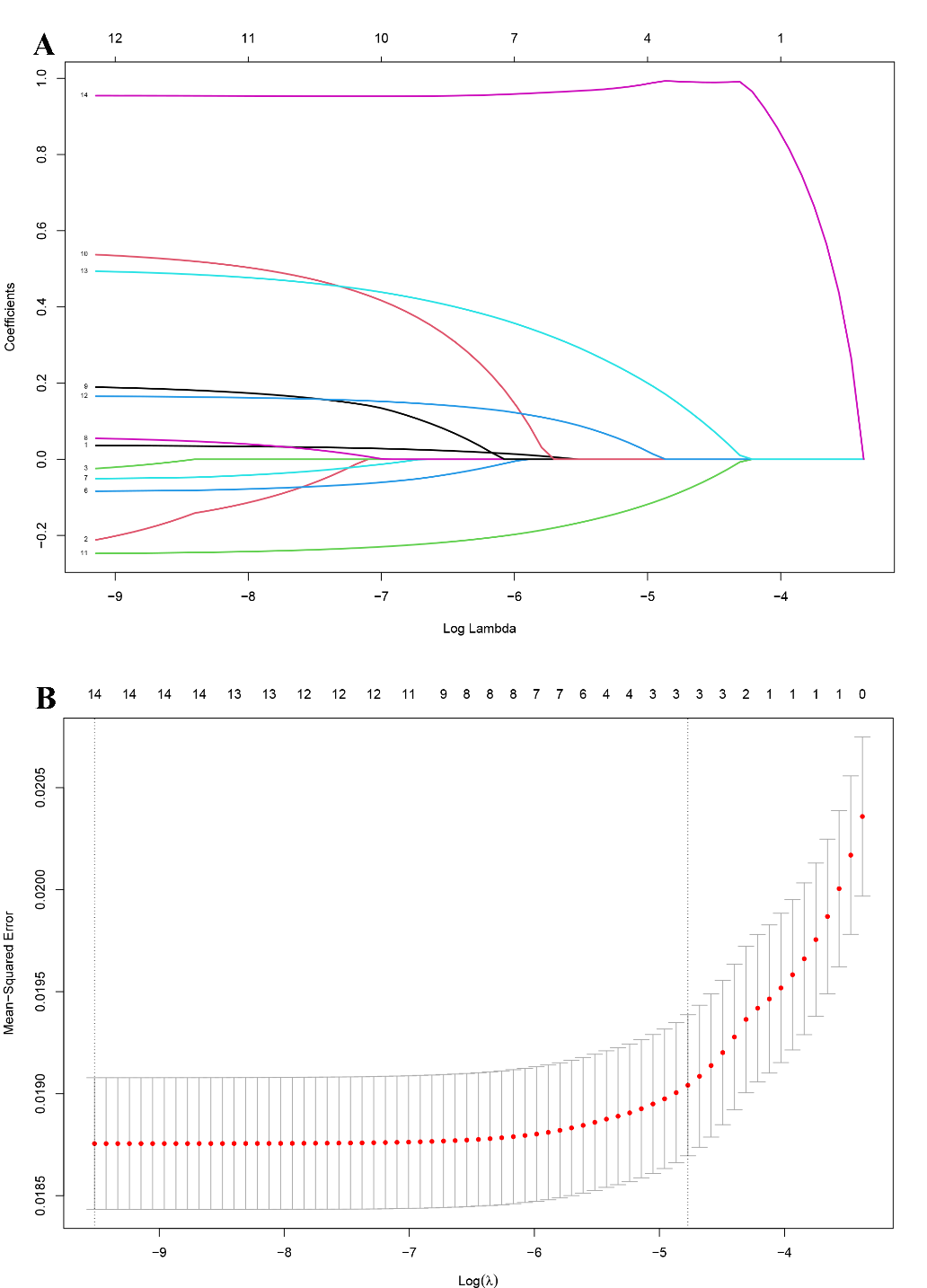


**Supplementary Figure S1.** The result of Lasso regression analysis of the multivariable model. (A) LASSO coefficients of 14 features; (B) Selection of tuning parameter (λ) for LASSO model.
